# Real-world database evaluation of drug-associated vitreous opacities and machine learning for clinical interpretability

**DOI:** 10.3389/fcell.2025.1699669

**Published:** 2026-01-12

**Authors:** Wenying Guan, Shi-Nan Wu, Ke Feng, Changsheng Xu, Yuwen Liu, Bing Yan, Jingyao Lv, Caihong Huang, Jiaoyue Hu, Zuguo Liu

**Affiliations:** 1 Xiamen University Affiliated Xiamen Eye Center, School of Medicine, Xiamen University, Xiamen, Fujian, China; 2 Fujian Provincial Key Laboratory of Ophthalmology and Visual Science, School of Medicine, Xiamen University, Xiamen, Fujian, China; 3 Fujian Engineering and Research Center of Eye Regenerative Medicine, School of Medicine, Xiamen University, Xiamen, Fujian, China; 4 Eye Institute of Xiamen University, School of Medicine, Xiamen University, Xiamen, Fujian, China; 5 Department of Ophthalmology, Xiang’an Hospital of Xiamen University, Xiamen, Fujian, China; 6 Department of Ophthalmology, The First Affiliated Hospital of University of South China, Hengyang, Hunan, China

**Keywords:** drug induction time, drug-induced risk, U.S. food and drug administration adverse event reporting system, machine learning, vitreous opacities

## Abstract

**Background:**

With visual disturbances from vitreous opacities (VOs) and floaters drawing increasing attention, we analyzed real-world data from the U.S. Food and Drug Administration Adverse Event Reporting System (FAERS) to characterize VO-associated drug profiles and inform clinical strategies for reducing VO-related complications.

**Materials and methods:**

Disproportionality analysis was performed on FAERS reports (2004–2024) to identify VO-associated drugs. Drugs were then classified to assess the onset time and baseline characteristics. Multivariable logistic regression was used to evaluate confounders. The predictive performance was compared using six machine learning algorithms, with SHapley Additive exPlanations (SHAP) used for feature importance.

**Results:**

Among 3,817 VO-related reports, 38 drugs were identified as independent risk factors, and they were mainly ocular, oncologic, hormonal, antimicrobial, and immunologic agents. Antimicrobial drugs had the earliest onset (mean 43.6 days), and hormonal drugs had the latest (mean 409.2 days). In the bootstrapped aggregating (BAG) model, the top predictors of VO were dexamethasone, reporter, time, brolucizumab, and age. The five highest-risk drugs were dexamethasone, brolucizumab, triamcinolone, faricimab, and fingolimod.

**Conclusion:**

This first systematic real-world evaluation of VO-related adverse drug reactions identifies high-risk drugs, susceptible populations, and onset patterns, thus offering guidance for preventive medication strategies. The BAG model showed higher sensitivity in real-world analysis, suggesting potential for further research in VO and floater prevention and treatment.

## Introduction

The vitreous is a transparent gel-like structure composed of approximately 98% water, along with macromolecules primarily consisting of collagen and hyaluronan (Bishop, 2000; Milston et al., 2016), non-collagenous proteins such as opticin and versican, and small amounts of trace metals (Ankamah et al., 2019). Collagen fibrils aggregate to form fibrous structures when dissociation occurs between collagen and hyaluronan. In addition, abnormal inflammatory responses, oxidative stress, reduced antioxidant capacity, increased free radicals, the presence of exogenous substances, and dysregulated collagenase activity can induce vitreous liquefaction (Font et al., 1978). These alterations project thread-like gray shadows containing dark spots or small nodules onto the retina, leading to the visual phenomenon known as floaters.

Vitreous opacities (VOs), as a benign and non-debilitating ocular condition, generally do not require special treatment and were largely overlooked before 2000 (Sun et al., 2025). However, with the progressive aging of the global population, the high prevalence of refractive errors, frequent use of visual display terminals (VDTs), increased public health awareness, and the advent of artificial intelligence, people have become increasingly aware that the prevalence of VO and floaters is much higher than previously recognized. Studies have found that approximately 76% of individuals are affected by vitreous floaters, with 33% of them reporting visual impairments such as blurred vision, difficulty driving at night, and difficulty recognizing faces (Webb et al., 2013; Milston et al., 2016). The negative impact of vitreous floaters on vision-related quality of life (Sebag et al., 2014; Castilla-Marti et al., 2015; Mamou et al., 2015; Senra et al., 2022; Paniagua-Diaz et al., 2024) even surpasses that of glaucoma and diabetic retinopathy (Wagle et al., 2011). Numerous studies have clearly demonstrated a strong association between vision loss, reduced quality of life, and mental health problems (Brown et al., 2000; McKean-Cowdin et al., 2010; Kempen et al., 2012; Taipale et al., 2019; Assi et al., 2021; Purola et al., 2021). Even mild visual impairments are significantly associated with a sustained decline in health-related quality of life. Among individuals under 55 years of age, up to 7% are willing to risk blindness in order to eliminate floaters [10, 11]. Furthermore, patients with higher intelligence and greater career achievements are more likely to seek active treatment (Sebag, 2020). Notably, current treatment options for VO and floaters are typically limited to conservative observation and follow-up, vitrectomy (Mason et al., 2014), or neodymium-doped yttrium aluminum garnet (Nd:YAG) laser therapy (van Bree et al., 2008). Conservative treatment mainly provides comfort and suggests active adaptation to this visual phenomenon, while surgical and laser treatments carry serious risks such as endophthalmitis, retinal tears, retinal detachment, cystoid macular edema, and macular pucker (Milston et al., 2016).

From a preventive perspective, researchers are exploring potential pathways to reduce the occurrence of VO and floaters by investigating their underlying causes. Previous studies have identified common causes of VO, including aging (Tozer et al., 2014), inflammation (Coupland, 2008), vitreoretinal dystrophy, myopic vitreopathy (Gale J. I. Y. and Sebag J., 2014), and diabetic vitreopathy (Gale J. A. L. and Sebag J., 2014), along with fundus vascular diseases, tumors, and trauma, all of which can lead to the entry of proteins, amyloid substances, or cells into the vitreous body. Myopia, allergic diseases, diabetes, and cardiovascular diseases all cause pathological changes in the local eye tissue and systemic health. In addition to the direct effects of these diseases on the vitreous body, the likelihood of requiring long-term medication to treat these conditions significantly increases. Local eye treatments, including eye drops, injections, and systemic medications, may affect the ocular tissues, blood vessels, and ocular microenvironment, potentially leading to or exacerbating the onset and progression of VO and floaters. Investigating the potential link between medications and VO or floaters holds direct clinical significance in reducing their occurrence during the course of disease treatment.

The FDA Adverse Event Reporting System (FAERS) database is widely used to collect real-world sample data on adverse events that occur during clinical practice and drug administration, and it is commonly employed to assess signals of drug-associated adverse reactions. This study utilizes the FAERS database, leveraging large-scale real-world data, to evaluate the occurrence of VO and floaters caused by medications. By optimizing the drug administration regimen for the treatment of this disease, the study aims to reduce the incidence of VO and floaters, thereby preventing additional visual disturbances and psychological burdens for patients. This approach is of significant value in improving patient adherence to treatment and enhancing the visual quality and overall quality of life during disease management.

## Methods

### Data source

**FIGURE 1:**
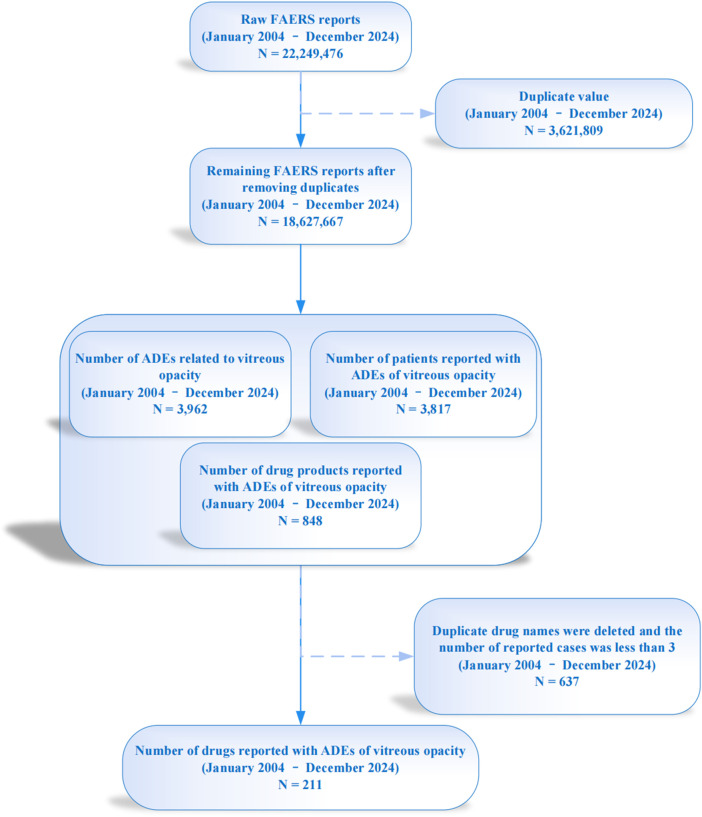
Data cleaning flowchart for drug-related vitreous opacity in the FAERS database from 2004 to 2024.

The data for this study were sourced from the FAERS database, covering the period from 1 January 2004 to 31 December 2024. The study data can be downloaded from the FDA website (https://fis.fda.gov/extensions/FPD-QDE-FAERS/FPD-QDE-FAERS.html). This database contains spontaneous adverse event reports from healthcare professionals, drug manufacturers, and drug users worldwide. Further details regarding the database can be found in our previous studies (Wu et al., 2024a; Wu et al., 2024b; Wu et al., 2025a; Wu et al., 2025b). To ensure the professionalism of the data sources, we specifically selected reports submitted by healthcare professionals, particularly physicians and pharmacists (coded as ‘MD,’ ‘PH,’ ‘OT,’ and ‘HP’). This allowed us to further analyze the adverse event incidence rates of all the medications in the database and the epidemiological characteristics of drug-related VO. From January 2004 to December 2024, the database contained a total of 22,249,476 raw records. After removing duplicate data based on the primary ID numbers, 18,627,667 records remained. Among these records, a total of 3,962 reports described adverse events related to VO, involving 3,817 subjects and 848 different medications associated with VO as an adverse reaction during drug use. Furthermore, we queried and replaced the generic and brand names of the drugs using the *DrugBank* database (https://go.drugbank.com/), excluding drugs with fewer than three reported cases (Wishart et al., 2018). Reports for drugs with different brand names but the same generic name were merged. Finally, 211 drugs were retained. A flowchart of the data cleaning process is shown in Figure 1.

### Identification of adverse drug reactions

The definition of adverse drug reactions analyzed in this study is derived from the 20.0 version of the Medical Dictionary for Regulatory Activities (MedDRA, http://www.meddra.org/) (Brown et al., 1999). Adverse drug reactions were coded using MedDRA® preferred terms (PTs), and standardized MedDRA® queries were used to identify PTs related to VO. In this study, we employed a ‘narrow’ scope of PTs (Kinoshita et al., 2020), which included ‘vitreous floaters,’ ‘VO,’ ‘myodesopsia,’ and ‘vitreous haze.’

### Statistical analysis

In this study, we employed four disproportionality analysis algorithms, including reporting odds ratio (ROR) (Rothman et al., 2004), proportional reporting ratio (PRR) (Evans et al., 2001), Bayesian confidence propagation neural network (BCPNN) (Bate, 2007), and multi-item gamma Poisson shrinkage (MGPS) (Wu et al., 2025a). These four methods were used to mine potential positive signals by comparing the target events and target drugs with all other events and drugs using a 2 × 2 contingency table calculation. The cell ‘a’ represents the number of occurrences of the target adverse event in the target drug, and detailed calculation methods can be found in Supplementary Tables S1 and S2. Additionally, we assessed the drug-related latency for VO in the intersection of positive signals identified by the four disproportionality analysis methods. Cumulative risk curves and univariate analysis of variance (ANOVA) were used to compare the latency periods of different drug types. Furthermore, to assess the influence of confounding factors such as age, gender, reporting country, drug administration route, and indications on positive signal drugs, we performed multivariable logistic regression analysis to evaluate drug risks. The diagnostic value of six different machine learning algorithms was compared by receiver operating characteristic (ROC) curves, and the best machine learning model was selected to quantify the feature importance of drug-related VO. Statistical analysis was conducted using Microsoft Excel 2021 and R (version 4.5.1), with *p* < 0.05 being considered statistically significant. During the R data analysis, the major packages used included ggplot2 (version 3.4.4), ggrepel (version 0.9.4), dplyr (version 1.1.4), forestploter (version 1.1.3), and DescTools (version 0.99.52). When building machine learning algorithms, we applied methods including adaptive boosting (Fei et al., 2023), logistic regression (Zhao et al., 2021), gradient boosting machine (GBM), extreme gradient boosting (XGB), multilayer perceptron (MLP), and bootstrapped aggregating (BAG) (Wu et al., 2024c). The core software program used in constructing these models was Python (version 3.11.13), with libraries such as sklearn (version 1.6.1), pandas (version 2.3.0), XGBoost (version 3.0.2), and NumPy (version 2.2.6).

## Results

### Baseline distribution of subjects

**FIGURE 2:**
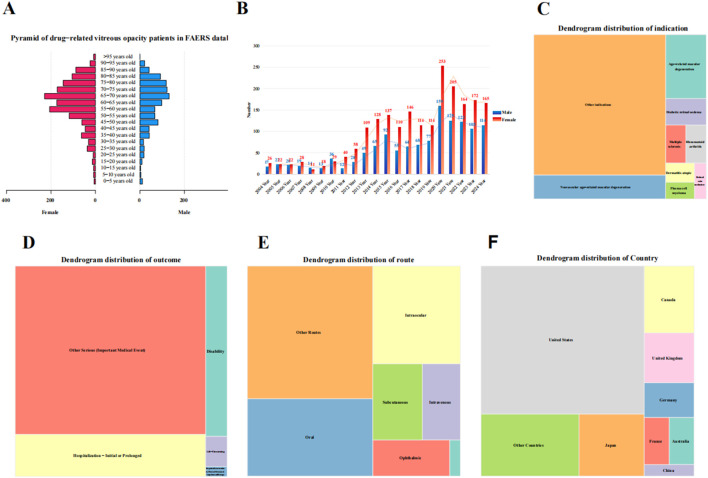
Baseline data distribution of subjects with drug-related vitreous opacity. **(A)** Age–gender pyramid. **(B)** Reporting trend of drug-related vitreous opacity from 2004 to 2024. **(C–F)** Dendrograms for the indications, outcomes, drug administration routes, and reporting countries, respectively.

This study included 3,817 subjects with adverse event reports related to VO. After data cleaning to exclude records with missing patient information, the average age of the subjects was 62.05 ± 17.58 years (mean ± standard deviation), with female subjects accounting for 61.88%. The age at which drug-related VO was reported was primarily concentrated between 65 and 70 years in both male and female subjects (Figure 2A). Since 2004, the number of reports related to drug-related VO has shown an increasing epidemiological trend, peaking in 2020, with a higher incidence in female subjects than in male subjects (Figure 2B). The distribution of indications was predominantly associated with neovascular age-related macular degeneration (nAMD) (11.13%) (Figure 2C), while the outcome distribution was mainly centered on hospitalization—initial or prolonged (17.69%) (Figure 2D). The most common route of administration was oral (21.69%) (Figure 2E), and the majority of reports came from the United States (53.92%) (Figure 2F). Most reports were submitted by physicians (59.92%). More details are provided in Figure 2 and Table 1.

**TABLE 1:** Baseline data distribution of drug-related vitreous opacity subjects.

Variable		Formula	Total
Age		Mean ± SD	62.05 ± 17.58
		Median (Q1, Q3)	65.00 (53.00, 75.00)
Weight		Mean ± SD	75.21 ± 20.94
		Median (Q1, Q3)	71.73 (61.00, 86.17)
Gender	Female	n (%)	2,071 (61.88)
	Male	n (%)	1,276 (38.12)
Reporter	Physician	n (%)	2,287 (59.92)
	Other health-professional	n (%)	664 (17.40)
	Health professional	n (%)	551 (14.44)
	Pharmacist	n (%)	315 (8.25)
Country	United States	n (%)	2,058 (53.92)
	Japan	n (%)	345 (9.04)
	Canada	n (%)	285 (7.47)
	United Kingdom	n (%)	212 (5.55)
	Germany	n (%)	148 (3.88)
	France	n (%)	101 (2.65)
	Australia	n (%)	100 (2.62)
	China	n (%)	49 (1.28)
	Other countries	n (%)	519 (13.60)
Route	Oral	n (%)	828 (21.69)
	Intraocular	n (%)	731 (19.15)
	Subcutaneous	n (%)	322 (8.44)
	Intravenous	n (%)	247 (6.47)
	Ophthalmic	n (%)	237 (6.21)
	Intramuscular	n (%)	33 (0.86)
	Other routes	n (%)	1,419 (37.18)
Outcome	Hospitalization—initial or prolonged	n (%)	484 (17.69)
	Disability	n (%)	221 (8.08)
	Death	n (%)	50 (1.83)
	Life-threatening	n (%)	39 (1.43)
	Required intervention to prevent permanent impairment/damage	n (%)	13 (0.48)
	Other serious (important medical event)	n (%)	1,929 (70.50)
Indication	Neovascular age-related macular degeneration	n (%)	425 (11.13)
	Age-related macular degeneration	n (%)	350 (9.17)
	Diabetic retinal edema	n (%)	145 (3.80)
	Multiple sclerosis	n (%)	104 (2.72)
	Rheumatoid arthritis	n (%)	104 (2.72)
	Dermatitis atopic	n (%)	75 (1.96)
	Plasma cell myeloma	n (%)	65 (1.70)
	Retinal vein occlusion	n (%)	57 (1.49)
	Other indications	n (%)	2,492 (65.29)

Continuous variables are presented as the mean ± standard deviation, while categorical variables are expressed as the sample size and percentage.

Abbreviations: SD, standard deviation.

### Drug distribution

**FIGURE 3:**
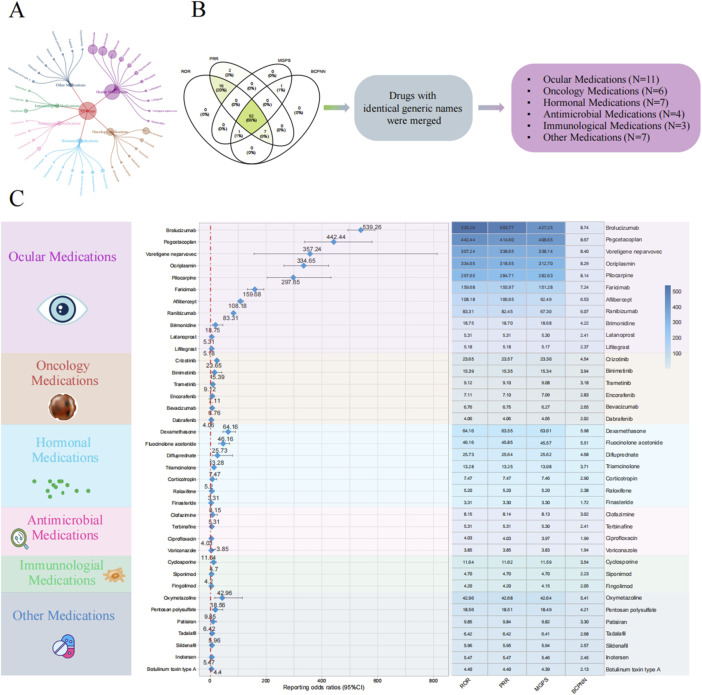
Disproportionality analysis for drug-related vitreous opacity. **(A)** Circular chart of adverse event reports for drug-related vitreous opacity categorized by drug type. **(B)** Screening process for the four disproportionality analysis algorithms. **(C)** Forest plot of ROR values for different drug categories and the heatmap of the results from the four disproportionality analysis algorithms. Abbreviations: BCPNN, Bayesian confidence propagation neural network; MGPS, multi-item gamma Poisson shrinker; PRR, proportional reporting ratio; ROR, reporting odds ratio; CI, confidence interval.

Based on the results of positive signals from the disproportionality analysis, we identified 38 drugs that were closely associated with VO. These drugs were primarily distributed across the following categories: ocular medications (N = 11), oncology medications (N = 6), hormonal medications (N = 7), antimicrobial medications (N = 4), immunological medications (N = 3), and other medications (N = 7). Among ocular medications, the drug most frequently reported for VO was ranibizumab (N = 737), while the drug with the highest ROR was brolucizumab, with an ROR (95% CI) of 539.26 (493.22–589.59). In the oncology category, the drug with the most reports of VO was bevacizumab (334 reports), and the drug with the highest ROR was crizotinib, with an ROR (95% CI) of 23.65 (17.78–31.46). For hormonal medications, the drug with the most reports of VO was triamcinolone (57 reports), and the drug with the highest ROR was dexamethasone, with an ROR (95% CI) of 64.16 (45.71–90.08). Among the four antimicrobial medications, ciprofloxacin had the highest number of reports of VO (78 reports), and the drug with the highest ROR was clofazimine, with an ROR (95% CI) of 8.15 (2.62–25.29). In the category of immunological medications, fingolimod had the most reports (63 reports), and the drug with the highest ROR was cyclosporine, with an ROR (95% CI) of 11.64 (6.44–21.05). Among the other medications, oxymetazoline had the highest number of reported VO adverse events (22 cases). Oxymetazoline also exhibited the highest ROR, with an ROR (95% confidence interval) of 42.96 (16.06–114.88). Further details are provided in Figure 3; Table 2.

**TABLE 2:** Positive signal results of drug-related vitreous opacity disproportionality analysis.

Drug	Drug category	ROR (95% CI)	PRR (95% CI)	MGPS (95% CI)	BCPNN (95% CI)	PRR (X^2^)	*p*-value
Brolucizumab	Ocular medications	539.26 (493.22–589.59)	503.77 (503.69–503.86)	427.25 (396.51–460.38)	8.74 (7.07–10.41)	503.77 (256559.85)	<0.001
Pegcetacoplan	Ocular medications	442.44 (337.74–579.59)	414.6 (414.35–414.85)	408.65 (326.01–512.25)	8.67 (7.01–10.34)	414.6 (23183.65)	<0.001
Voretigene neparvovec	Ocular medications	357.24 (156.95–813.13)	338.65 (337.87–339.43)	338.14 (169.91–672.95)	8.4 (6.71–10.09)	338.65 (2017.19)	<0.001
Ocriplasmin	Ocular medications	334.65 (263.97–424.25)	318.55 (318.33–318.78)	312.7 (256.4–381.36)	8.29 (6.62–9.96)	318.55 (22686.3)	<0.001
Pilocarpine	Ocular medications	297.65 (204.88–432.41)	284.71 (284.35–285.07)	282.63 (206.78–386.31)	8.14 (6.47–9.81)	284.71 (8139.92)	<0.001
Faricimab	Ocular medications	159.68 (132.87–191.89)	155.97 (155.79–156.15)	151.28 (129.72–176.43)	7.24 (5.57–8.91)	155.97 (17920.68)	<0.001
Aflibercept	Ocular medications	108.18 (98.68–118.61)	106.65 (106.56–106.75)	92.49 (85.64–99.89)	6.53 (4.86–8.2)	106.65 (48135.52)	<0.001
Ranibizumab	Ocular medications	83.31 (76.88–90.28)	82.45 (82.38–82.53)	67.3 (62.93–71.98)	6.07 (4.41–7.74)	82.45 (48279.96)	<0.001
Brimonidine	Ocular medications	18.75 (7.79–45.13)	18.7 (17.82–19.58)	18.68 (8.96–38.95)	4.22 (2.56–5.89)	18.7 (83.67)	<0.001
Latanoprost	Ocular medications	5.31 (2.86–9.89)	5.31 (4.69–5.93)	5.3 (3.15–8.91)	2.41 (0.74–4.07)	5.31 (34.91)	<0.001
Lifitegrast	Ocular medications	5.18 (2.47–10.89)	5.18 (4.44–5.92)	5.17 (2.78–9.62)	2.37 (0.7–4.04)	5.18 (23.58)	<0.001
Crizotinib	Oncology medications	23.65 (17.78–31.46)	23.57 (23.29–23.86)	23.3 (18.35–29.58)	4.54 (2.88–6.21)	23.57 (1025.03)	<0.001
Binimetinib	Oncology medications	15.39 (5.77–41.07)	15.35 (14.37–16.33)	15.34 (6.75–34.88)	3.94 (2.27–5.61)	15.35 (53.63)	<0.001
Trametinib	Oncology medications	9.12 (5.29–15.72)	9.1 (8.56–9.65)	9.08 (5.75–14.32)	3.18 (1.52–4.85)	9.1 (93.48)	<0.001
Encorafenib	Oncology medications	7.11 (3.39–14.93)	7.1 (6.36–7.84)	7.09 (3.81–13.19)	2.83 (1.16–4.49)	7.1 (36.65)	<0.001
Bevacizumab	Oncology medications	6.76 (6.04–7.56)	6.75 (6.64–6.86)	6.27 (5.71–6.88)	2.65 (0.98–4.31)	6.75 (1499.18)	<0.001
Dabrafenib	Oncology medications	4.06 (2.25–7.34)	4.06 (3.47–4.65)	4.05 (2.47–6.64)	2.02 (0.35–3.68)	4.06 (25.28)	<0.001
Dexamethasone	Hormonal medications	64.16 (45.71–90.08)	63.55 (63.21–63.89)	63.01 (47.44–83.7)	5.98 (4.31–7.64)	63.55 (2075.58)	<0.001
Fluocinolone acetonide	Hormonal medications	46.16 (30.86–69.06)	45.85 (45.45–46.25)	45.57 (32.54–63.84)	5.51 (3.84–7.18)	45.85 (1046.61)	<0.001
Difluprednate	Hormonal medications	25.73 (8.28–80)	25.64 (24.51–26.77)	25.62 (9.92–66.18)	4.68 (3.01–6.35)	25.64 (70.98)	<0.001
Triamcinolone	Hormonal medications	13.28 (10.22–17.25)	13.25 (12.99–13.52)	13.08 (10.51–16.28)	3.71 (2.04–5.38)	13.25 (636.62)	<0.001
Corticotropin	Hormonal medications	7.47 (2.41–23.2)	7.47 (6.33–8.6)	7.46 (2.89–19.25)	2.9 (1.23–4.57)	7.47 (16.79)	0.001
Raloxifene	Hormonal medications	5.2 (1.95–13.88)	5.2 (4.22–6.18)	5.2 (2.29–11.81)	2.38 (0.71–4.04)	5.2 (13.56)	0.002
Finasteride	Hormonal medications	3.31 (1.92–5.7)	3.3 (2.76–3.85)	3.3 (2.09–5.2)	1.72 (0.05–3.39)	3.3 (20.83)	<0.001
Clofazimine	Antimicrobial medications	8.15 (2.62–25.29)	8.14 (7.01–9.27)	8.13 (3.15–20.98)	3.02 (1.36–4.69)	8.14 (18.77)	<0.001
Terbinafine	Antimicrobial medications	5.31 (2.38–11.83)	5.31 (4.51–6.11)	5.3 (2.71–10.36)	2.41 (0.74–4.07)	5.31 (20.94)	<0.001
Ciprofloxacin	Antimicrobial medications	4.03 (3.22–5.05)	4.03 (3.81–4.26)	3.97 (3.29–4.79)	1.99 (0.32–3.66)	4.03 (174.44)	<0.001
Voriconazole	Antimicrobial medications	3.85 (2.62–5.66)	3.85 (3.46–4.23)	3.83 (2.77–5.28)	1.94 (0.27–3.6)	3.85 (54.39)	<0.001
Cyclosporine	Immunological medications	11.64 (6.44–21.05)	11.62 (11.03–12.21)	11.59 (7.06–19.03)	3.54 (1.87–5.2)	11.62 (106.5)	<0.001
Siponimod	Immunological medications	4.7 (1.96–11.31)	4.7 (3.82–5.58)	4.7 (2.25–9.78)	2.23 (0.56–3.9)	4.7 (14.55)	0.001
Fingolimod	Immunological medications	4.2 (3.28–5.39)	4.2 (3.95–4.45)	4.15 (3.37–5.11)	2.05 (0.39–3.72)	4.2 (151.26)	<0.001
Oxymetazoline	Other medications	42.96 (16.06–114.88)	42.68 (41.7–43.66)	42.64 (18.72–97.11)	5.41 (3.74–7.08)	42.68 (162.68)	<0.001
Pentosan polysulfate	Other medications	18.56 (7.71–44.67)	18.51 (17.63–19.38)	18.49 (8.86–38.55)	4.21 (2.54–5.88)	18.51 (82.72)	<0.001
Patisiran	Other medications	9.85 (4.42–21.96)	9.84 (9.04–10.64)	9.82 (5.02–19.21)	3.3 (1.63–4.96)	9.84 (47.57)	<0.001
Tadalafil	Other medications	6.42 (2.67–15.45)	6.42 (5.54–7.3)	6.41 (3.08–13.36)	2.68 (1.01–4.35)	6.42 (22.85)	<0.001
Sildenafil	Other medications	5.96 (3.1–11.46)	5.95 (5.3–6.61)	5.94 (3.44–10.27)	2.57 (0.9–4.24)	5.95 (37.02)	<0.001
Inotersen	Other medications	5.47 (2.73–10.95)	5.47 (4.77–6.16)	5.46 (3.05–9.75)	2.45 (0.78–4.11)	5.47 (29.14)	<0.001
Botulinum toxin type A	Other medications	4.4 (2.5–7.76)	4.4 (3.83–4.97)	4.39 (2.73–7.05)	2.13 (0.47–3.8)	4.4 (31.42)	<0.001

The *p*-value represents the statistical test value from the chi-square test in the PRR algorithm. All of the above drugs meet the positive signal screening criteria for disproportionality analysis.

Abbreviations: BCPNN, Bayesian confidence propagation neural network; MGPS, multi-item gamma Poisson shrinker; PRR, proportional reported ratio; ROR, reporting odds ratio; CI, confidence interval.

### Comparison of drug-induced time

**FIGURE 4:**
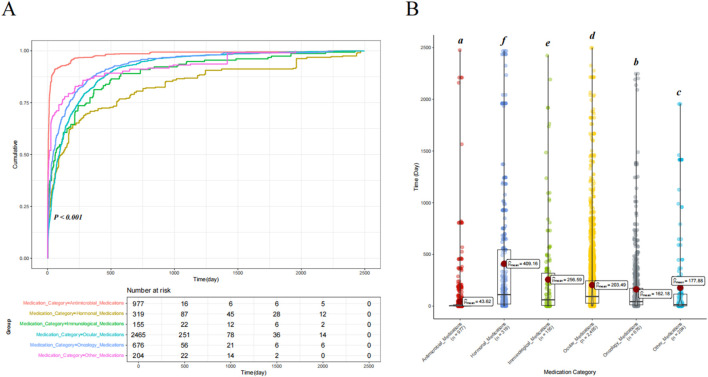
Comparison of the cumulative risk curves and variance analysis results for drug-induced time of occurrence across different drug categories. **(A)** Comparison of the cumulative risk curves for drug-induced time of occurrence across different drug categories. **(B)** Comparison of the univariate variance analysis results for drug-induced time of occurrence across different drug categories. Letter notation is used for labeling.

**FIGURE 5:**
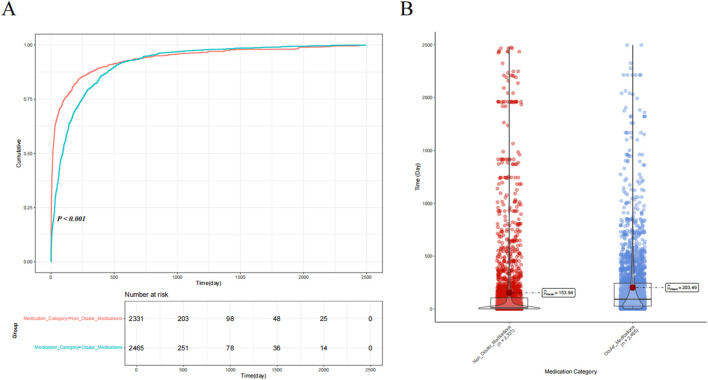
Comparison of drug-induced time of occurrence between ocular medications and non-ocular medications. **(A)** Comparison of the cumulative risk curves for ocular and non-ocular medications. **(B)** Results of an independent samples t-test between ocular and non-ocular medications.

We classified the drugs into six categories based on their mechanisms of action, namely, ocular medications, oncology medications, hormonal medications, antimicrobial medications, immunological medications, and other drugs, to assess differences in the time to onset of drug-related VO and floaters. Cumulative risk curve analysis showed significant differences in the time to onset of drug-related VO among the different drug categories (*p* < 0.001). Furthermore, ANOVA revealed that antimicrobial medications had the shortest onset time (mean = 43.62 days), while hormonal medications had the longest (mean = 409.16 days) (see Figure 4). When drugs were classified as ocular or non-ocular medications, the cumulative risk curve also showed significant differences in the onset time between the two groups, with ocular medications having a significantly longer onset time than non-ocular medications (203.49 days vs. 153.94 days) (*p* < 0.001) (see Figure 5).

### Multivariate analysis results

**FIGURE 6:**
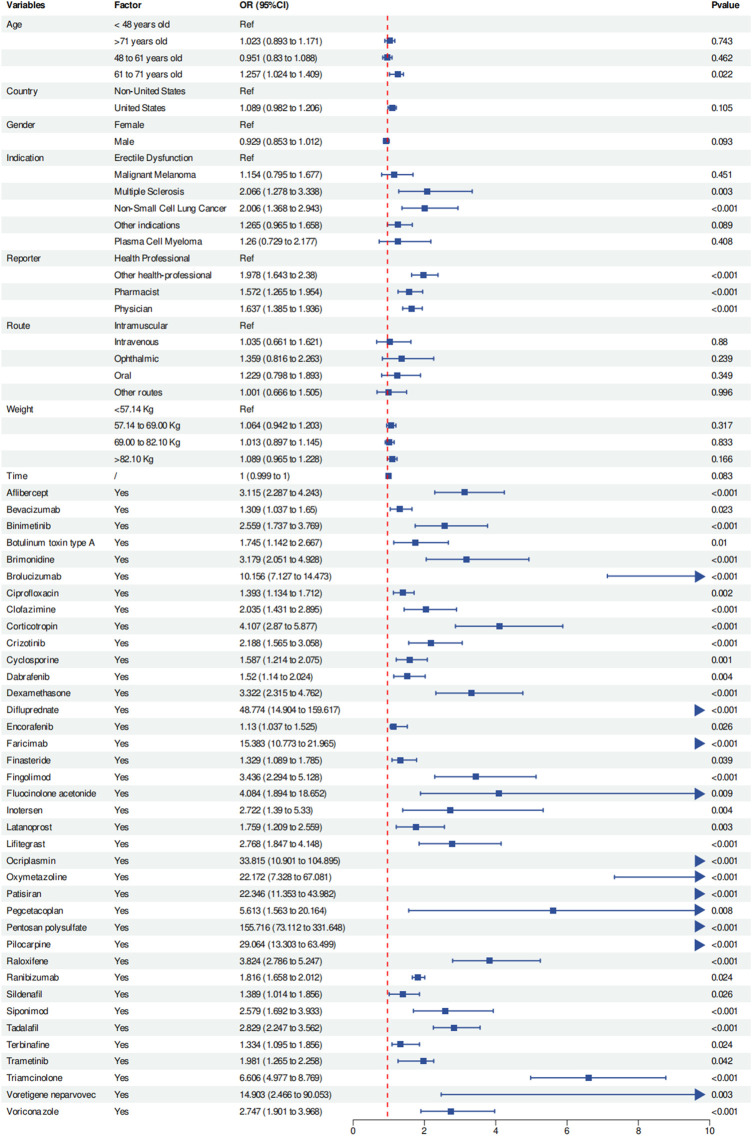
Multivariate logistic regression analysis results. Abbreviations: OR, odds ratio; CI, confidence interval.

We included relevant data for all subjects associated with the 38 drugs—such as age, weight, gender, country, indications, reporter type, route of administration, and duration of drug use—for multivariate logistic regression analysis. The results showed that all 38 drugs were independent risk factors for drug-related VO (OR and 95% CI > 1). Age between 61 and 71 years was found to be a relative risk factor for developing VO compared to those under 48 years [OR (95% CI) = 1.257 (1.024–1.409)]. Furthermore, subjects with multiple sclerosis [OR (95% CI) = 2.066 (1.278–3.338)] and non-small cell lung cancer [OR (95% CI) = 2.006 (1.368–2.943)] had a higher risk of developing VO than those with erectile dysfunction (*p* < 0.05). Among the reporters, ‘other health-professionals’ [OR (95% CI) = 1.978 (1.643–2.38)], ‘pharmacists’ [OR (95% CI) = 1.572 (1.265–1.954)], and ‘physicians’ [OR (95% CI) = 1.637 (1.385–1.936)] reported a higher risk of VO than ‘health professionals’ (*p* < 0.05). Further details can be found in Figure 6; Table 3.

**TABLE 3:** Results of multivariate logistic regression analysis on drug-related vitreous opacity.

Variable	Factor	OR (95% CI)	*p*-value
Age	<48 years old	Reference	Reference
	>71 years old	1.023 (0.893–1.171)	0.743
	48–61 years old	0.951 (0.83–1.088)	0.462
	61–71 years old	1.257 (1.024–1.409)	0.022
Country	Non-United States	Reference	Reference
	United States	1.089 (0.982–1.206)	0.105
Gender	Female	Reference	Reference
	Male	0.929 (0.853–1.012)	0.093
Indication	Erectile dysfunction	Reference	Reference
	Malignant melanoma	1.154 (0.795–1.677)	0.451
	Multiple sclerosis	2.066 (1.278–3.338)	0.003
	Non-small-cell lung cancer	2.006 (1.368–2.943)	<0.001
	Other indications	1.265 (0.965–1.658)	0.089
	Plasma cell myeloma	1.26 (0.729–2.177)	0.408
Reporter	Health professional	Reference	Reference
	Other health-professional	1.978 (1.643–2.38)	<0.001
	Pharmacist	1.572 (1.265–1.954)	<0.001
	Physician	1.637 (1.385–1.936)	<0.001
Route	Intramuscular	Reference	Reference
	Intravenous	1.035 (0.661–1.621)	0.88
	Ophthalmic	1.359 (0.816–2.263)	0.239
	Oral	1.229 (0.798–1.893)	0.349
	Other routes	1.001 (0.666–1.505)	0.996
Weight	<57.14 Kg	Reference	Reference
	57.14 Kg–69.00 Kg	1.064 (0.942–1.203)	0.317
	69.00 Kg–82.10 Kg	1.013 (0.897–1.145)	0.833
	>82.10 Kg	1.089 (0.965–1.228)	0.166
Time	—	1 (0.999–1)	0.083
Aflibercept	No	Reference	Reference
	Yes	3.115 (2.287–4.243)	<0.001
Bevacizumab	No	Reference	Reference
	Yes	1.309 (1.037–1.65)	0.023
Binimetinib	No	Reference	Reference
	Yes	2.559 (1.737–3.769)	<0.001
Botulinum_toxin_type_A	No	Reference	Reference
	Yes	1.745 (1.142–2.667)	0.01
Brimonidine	No	Reference	Reference
	Yes	3.179 (2.051–4.928)	<0.001
Brolucizumab	No	Reference	Reference
	Yes	10.156 (7.127–14.473)	<0.001
Ciprofloxacin	No	Reference	Reference
	Yes	1.393 (1.134–1.712)	0.002
Clofazimine	No	Reference	Reference
	Yes	2.035 (1.431–2.895)	<0.001
Corticotropin	No	Reference	Reference
	Yes	4.107 (2.87–5.877)	<0.001
Crizotinib	No	Reference	Reference
	Yes	2.188 (1.565–3.058)	<0.001
Cyclosporine	No	Reference	Reference
	Yes	1.587 (1.214–2.075)	0.001
Dabrafenib	No	Reference	Reference
	Yes	1.52 (1.14–2.024)	0.004
Dexamethasone	No	Reference	Reference
	Yes	3.322 (2.315–4.762)	<0.001
Difluprednate	No	Reference	Reference
	Yes	48.774 (14.904–159.617)	<0.001
Encorafenib	No	Reference	Reference
	Yes	1.13 (1.037–1.525)	0.026
Faricimab	No	Reference	Reference
	Yes	15.383 (10.773–21.965)	<0.001
Finasteride	No	Reference	Reference
	Yes	1.329 (1.089–1.785)	0.039
Fingolimod	No	Reference	Reference
	Yes	3.436 (2.294–5.128)	<0.001
Fluocinolone_acetonide	No	Reference	Reference
	Yes	4.084 (1.894–18.652)	0.009
Inotersen	No	Reference	Reference
	Yes	2.722 (1.39–5.33)	0.004
Latanoprost	No	Reference	Reference
	Yes	1.759 (1.209–2.559)	0.003
Lifitegrast	No	Reference	Reference
	Yes	2.768 (1.847–4.148)	<0.001
Ocriplasmin	No	Reference	Reference
	Yes	33.815 (10.901–104.895)	<0.001
Oxymetazoline	No	Reference	Reference
	Yes	22.172 (7.328–67.081)	<0.001
Patisiran	No	Reference	Reference
	Yes	22.346 (11.353–43.982)	<0.001
Pegcetacoplan	No	Reference	Reference
	Yes	5.613 (1.563–20.164)	0.008
Pentosan_polysulfate	No	Reference	Reference
	Yes	155.716 (73.112–331.648)	<0.001
Pilocarpine	No	Reference	Reference
	Yes	29.064 (13.303–63.499)	<0.001
Raloxifene	No	Reference	Reference
	Yes	3.824 (2.786–5.247)	<0.001
Ranibizumab	No	Reference	Reference
	Yes	1.816 (1.658–2.012)	0.024
Sildenafil	No	Reference	Reference
	Yes	1.389 (1.014–1.856)	0.026
Siponimod	No	Reference	Reference
	Yes	2.579 (1.692–3.933)	<0.001
Tadalafil	No	Reference	Reference
	Yes	2.829 (2.247–3.562)	<0.001
Terbinafine	No	Reference	Reference
	Yes	1.334 (1.095–1.856)	0.024
Trametinib	No	Reference	Reference
	Yes	1.981 (1.265–2.258)	0.042
Triamcinolone	No	Reference	Reference
	Yes	6.606 (4.977–8.769)	<0.001
Voretigene_neparvovec	No	Reference	Reference
	Yes	14.903 (2.466–90.053)	0.003
Voriconazole	No	Reference	Reference
	Yes	2.747 (1.901–3.968)	<0.001

Multivariate logistic regression analysis was performed on the drugs with positive disproportionality signals, considering variables such as age, gender, reporting country, reporter, indication, drug administration method, and weight.

Abbreviations: OR, odds ratio; CI, confidence interval.

### Comparison of the machine learning models and SHAP quantification

**FIGURE 7:**
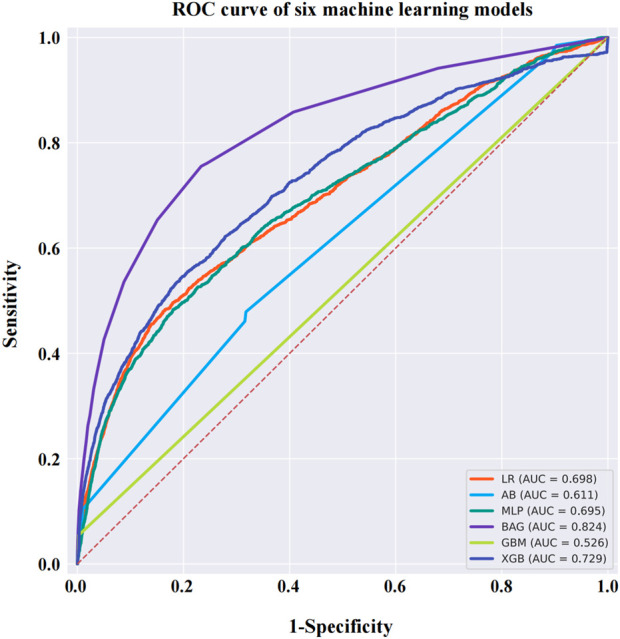
ROC curves for six machine learning models. The BAG model has a higher AUC value than the other models, reaching 0.824. Abbreviations: ROC, receiver operating characteristic curve; AB, adaptive boosting; LR, logistic regression; BAG, bootstrapped aggregating; MLP, multilayer perceptron; GBM, gradient boosting machine; XGB, extreme gradient boosting.

**FIGURE 8:**
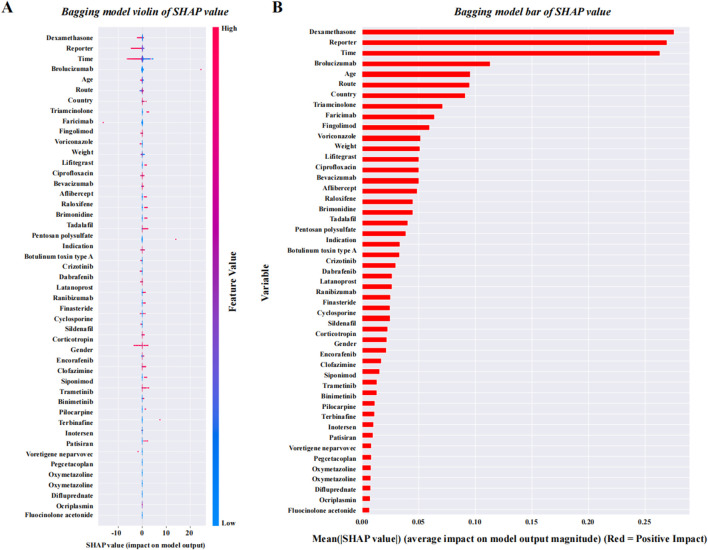
SHAP quantification results of the BAG model. **(A)** SHAP violin plot for the BAG model, which is ranked from high to low. **(B)** SHAP bar values for the BAG model, which are ranked from high to low. Abbreviations: SHAP, SHapley Additive exPlanations.

After data cleaning, we constructed six machine learning models with VO occurrence as the dependent variable. The BAG model outperformed the LR model, with AUC values of 0.824 and 0.698, respectively. The DeLong test confirmed a statistically significant difference between the two models (*p* < 0.001) (Figure 7). SHAP was applied to the BAG model to quantify the contribution of each variable to VO occurrence. The top five contributing features were dexamethasone, reporter, time, brolucizumab, and age. Among the 38 drugs, the top five contributors were dexamethasone, brolucizumab, triamcinolone, faricimab, and fingolimod. Detailed results can be found in Figure 8.

## Discussion

VO and floaters had long been considered ‘a normal aging phenomenon,’ but after 2000, they began to receive increased attention from the medical community due to increasing patient complaints and advancements in imaging techniques (Sun et al., 2025). Currently, the contradiction between limited treatment options (Le Rouic et al., 2011; Schulz-Key et al., 2011; Tan et al., 2011; Khoshnevis and Sebag, 2015) and the growing clinical demand for treatment has become a pressing issue in this field. One potential solution is to reduce and prevent the occurrence of VO by addressing controllable factors. This study, based on real-world data from the FAERS database, is the first to focus on the occurrence of VO and floaters during clinical medication use. It provides a detailed analysis of 38 potential high-risk drugs associated with drug-related VO, compares the onset time across different drug categories, evaluates the impact of various confounding factors on drug-related VO, and builds six different machine learning models to identify the best-performing BAG model for evaluating the importance of each feature.

In this study, we conducted an analysis of adverse drug events using data from the FAERS database spanning from 1 January 2004 to 31 December 2024. We found that the analysis of baseline characteristics of the subjects, including the age at occurrence of drug-related VO, the epidemiological trend of case reports, and the range of drug indications involved, was consistent with previous research on VO (Milston et al., 2016; Sun et al., 2025). The baseline analysis of the drug-related VO population revealed a higher prevalence in female subjects than in male subjects. This finding aligns with the observation that the primary indications for drugs associated with VO were predominantly related to nAMD. Previous studies have shown that the incidence of nAMD is slightly higher in female subjects than in male subjects (Klein et al., 1999; VanNewkirk et al., 2000). This is related to the longer life expectancy in female subjects, along with the effects of estrogen withdrawal on choroidal neovascularization through mechanisms involving oxidative stress and VEGF regulation (Hardy et al., 2000).

We found that the distribution of drugs associated with VO was primarily concentrated in six categories: ocular medications, oncology medications, hormonal medications, antimicrobial medications, immunological medications, and other drugs. Among these, other drugs, such as muscarinic receptor agonists and botulinum toxin type-A, carry a higher risk. Although the drugs associated with VO appear diverse at first glance, further analysis revealed that they are primarily used by individuals over the age of 50, with indications primarily related to chronic diseases. The mechanisms of action of these drugs primarily involve inflammation, immunity, angiogenesis, and the regulation of intraocular pressure. These characteristics are consistent with the logic of research on the pathogenic factors of VO (Milston et al., 2016), thus providing supporting evidence for clinical medication choices based on real-world data.

The results from the BAG model show that the top five drugs associated with VO are dexamethasone, brolucizumab, triamcinolone, faricimab, and fingolimod. Further analysis revealed that four of these drugs (dexamethasone, brolucizumab, triamcinolone, and faricimab) are primarily used in the treatment of retinal diseases, with indications mainly involving macular edema, age-related macular degeneration, diabetic macular edema, retinal vein occlusion, and other ocular diseases. The patients are typically middle-aged or elderly individuals with diabetes or retinal degenerative diseases. Except for fingolimod, the four other drugs are administered directly into the eye via intravitreal injection. The mechanisms of action for all five drugs are primarily anti-inflammatory and anti-angiogenic. Although fingolimod is a systemic immunomodulator, its impact on ocular autoimmune conditions (such as uveitis) has also raised concern (Lim et al., 2019). During clinical practice, there is a trend of alternating use among some of these drugs. Based on the above analysis, we further considered the potential contribution of the route of administration to drug-related VO. However, due to missing data on administration routes in the FAERS database and the lack of clarity regarding the use of intravitreal injections, these results require further validation. Similarly, the shared mechanisms of action of VO-associated drugs, particularly their anti-inflammatory and anti-angiogenic effects, also warrant attention, as they may offer insights into future scientific research directions related to VO treatment. The study results indicate that there are differences in the time required for the occurrence of drug-related VO and floaters, but they all occur after long-term medication use (the shortest time being 43.62 days), suggesting that there is no need for excessive concern over short-term use of the high-risk drugs listed in this study during clinical treatment.

However, this study still has several limitations. First, although it explores the association between drugs and VO-related adverse events, it cannot establish a causal relationship. Second, as a spontaneous and voluntary reporting system, the FAERS database is inherently susceptible to reporting bias and incomplete data. On the one hand, the reported information may be influenced by the reporter’s subjective judgment, leading to potential inconsistencies and underreporting. On the other hand, from a clinical research design perspective, the vitreous condition prior to drug exposure should ideally be included as a baseline indicator for analysis. We reviewed the data and confirmed that such information is largely unavailable in the FAERS database due to its intrinsic nature as a post-marketing surveillance system rather than a cohort-based clinical dataset. Although FAERS records demographic characteristics, reporting time and source, drug information, adverse event descriptions, patient outcomes, and diagnoses, it lacks detailed ophthalmic examination findings and ocular history. Therefore, objective baseline vitreous conditions before drug administration cannot be reliably determined. Third, and importantly, the FAERS database does not contain standardized grading or quantitative indicators reflecting the severity or degree of VO. This limitation precludes us from comparing the severity of VO induced by different drugs. Any attempt to infer severity from descriptive narratives would introduce considerable bias due to the absence of consistent reporting standards. Consequently, we have explicitly acknowledged the “lack of severity grading information” as a limitation in this study. Future prospective clinical studies should employ standardized clinical grading systems—such as quantitative VO scoring scales—to collect severity data, thereby enabling more clinically meaningful comparisons across drug categories.

## Conclusion

This is the first study to systematically evaluate adverse reactions of systemic medications associated with VO and floaters using real-world data. It identifies medication-sensitive populations, drug types, and treatment durations linked to these events, providing a reference for clinicians to adjust regimens from a preventive perspective. The study also confirms that the BAG model offers greater sensitivity in real-world data analysis. These findings suggest potential research directions for the prevention and treatment of VO and floaters.
